# Experimental Study on the Shear Transfer Resistance of Uncracked Concrete Interfaces Reinforced with GFRP

**DOI:** 10.3390/polym18192357

**Published:** 2026-09-27

**Authors:** Ahmed A. Alimran, Ahmed K. El-Sayed, Mshtaq Ahmed

**Affiliations:** Department of Civil Engineering, College of Engineering, King Saud University, Riyadh 12372, Saudi Arabia; mshahmed@ksu.edu.sa

**Keywords:** GFRP reinforcement, interface, monolithic uncracked concrete, push-off test, residual strength, shear transfer capacity

## Abstract

The shear transfer capacity of initially uncracked concrete push-off specimens reinforced with glass-fiber-reinforced polymer (GFRP) stirrups is experimentally evaluated and presented in this paper. The specimens were cast monolithically using the same concrete mix and tested under monotonic loading conditions until failure. The test matrix included eight specimens with GFRP clamping reinforcement crossing the shear transfer plane, three specimens with steel clamping reinforcement, and one control specimen without clamping reinforcement. The test variables were the type (steel and GFRP) and the amount of clamping reinforcement, with reinforcement ratios ranging from 0.28% to 1.9%. The variation in GFRP reinforcement ratio was achieved either by changing the number of clamping stirrups crossing the shear transfer plane while maintaining the same bar size, or by varying the bar size while keeping the number of stirrups constant. The test results showed that increasing the clamping reinforcement ratio led to higher ultimate and residual shear transfer strengths. However, this trend was not observed for the cracking strength of the specimens. The results also indicated that GFRP-reinforced specimens exhibited lower strength compared with those reinforced with steel reinforcement due to the lower modulus of elasticity of GFRP. The ultimate interface shear strength of the GFRP-reinforced specimens ranged from 5.69 to 9.13 MPa, compared with 6.55 to 10.96 MPa for the steel-reinforced specimens. All reinforced specimens exhibited a similar mode of failure, characterized by shear failure along the interface plane. The analyzed results demonstrated that the stiffness of the clamping stirrups is a key parameter affecting the interface shear behavior. However, it should not be considered the sole governing factor, as the stirrup distribution and bar diameter also influenced the shear behavior. The shear transfer capacities of the GFRP-reinforced specimens were predicted using the AASHTO LRFD, CSA S6, and Vega and others models and compared with the experimental results. The comparison showed notable differences in the accuracy, conservatism, and consistency of the three models in predicting the experimental shear resistance.

## 1. Introduction

The transfer of shear stresses across concrete interfaces is a key factor governing the performance of various structural systems. Such interfaces may include existing cracks, potential crack planes, or construction joints formed between concrete cast at different times (cold joints). The transfer of shear stresses along these interfaces is commonly referred to as aggregate interlock, interface shear transfer, or shear friction [1]. The effectiveness of this transfer mechanism has a direct influence on structural strength, serviceability performance, and long-term durability. The mechanism of shear resistance across concrete interfaces occurs through the combined action of several interacting components. Cohesion or adhesion, as the initial component, provides a chemical bond between the contacting surfaces. As the relative displacement between the two sides of the interface increases, aggregate interlock is mobilized and contributes to the shear resistance. Providing clamping reinforcement across the interface restrains crack widening, maintaining the close contact required for effective aggregate interlock [2]. As the reinforcement is engaged in tension, it generates a compressive clamping force across the interface, thereby developing the frictional component and increasing the overall shear resistance of the interface. If an external compressive force exists and is applied perpendicular to the interface plane, it also contributes to the frictional component. At higher levels of relative displacement, dowel action provided by the clamping reinforcement becomes the dominant component contributing to shear transfer.

Research on the shear transfer behavior of concrete has been a topic of interest in structural engineering since the 1960s with the use of steel reinforcement as clamping reinforcement across interfaces. Early pioneering studies [3,4,5,6,7] advanced the understanding of shear transfer performance in reinforced concrete. Building on these studies, together with subsequent research [8,9,10,11], the shear friction hypothesis was applied, calibrated, and incorporated into most international design codes [12,13,14,15,16]. However, the long-term performance of steel reinforcement may be adversely affected in corrosive environments, which has encouraged the exploration of alternative reinforcement materials with improved durability. Among these alternatives, glass-fiber-reinforced polymer (GFRP) reinforcement has attracted growing interest due to its resistance to corrosion, relatively low density, and high tensile strength [17]. These advantageous properties make GFRP reinforcement uniquely suited for concrete infrastructure in harsh operating environments, such as marine and coastal facilities, bridge decks exposed to deicing salts, and parking garages, where steel corrosion drives premature structural deterioration. The use of GFRP reinforcement has been demonstrated to provide good durability in alkaline concrete environments, although its long-term performance depends on the characteristics of the fiber, polymer matrix, and manufacturing process [18,19]. Nevertheless, GFRP bars exhibit linear–elastic behavior up to rupture, without yielding, and have a lower modulus of elasticity than steel. These fundamental differences raise questions about the applicability of conventional shear-friction models when GFRP reinforcement is used, as such models rely on the assumption of yielding behavior and the development of clamping forces through bar deformation. In response to this gap, recent investigations have been conducted to characterize the shear transfer behavior when GFRP is used as clamping reinforcement [20,21,22,23,24,25,26,27]. Nevertheless, the available research is still limited and does not fully address all aspects relevant to the design of GFRP for shear friction applications.

The experimental assessment of shear transfer is commonly performed using push-off specimens composed of two interlocking L-shaped concrete blocks, as shown in Figure 1. This configuration enables isolation of the interface and the application of direct shear along a predefined plane. Depending on the condition of the interface prior to the application of shear load, three typical cases are available in the literature: monolithic uncracked interfaces, monolithic pre-cracked interfaces, and cold-jointed interfaces. These cases represent the range of conditions encountered in practice. Accordingly, conventional shear friction models are applied to various types of concrete interfaces to account for these situations. In fact, most of the limited studies available have focused on initially pre-cracked or cold-jointed specimens, while comparatively few have investigated initially uncracked specimens. Connor and Kim [20] tested initially pre-cracked push-off specimens to evaluate the interface shear performance with GFRP clamping reinforcement of varying orientations. Their results showed that crack width, slip, and the orientation of GFRP are important parameters for predicting the interface shear capacity. On the other hand, studies on cold-jointed specimens [21,22,23,24,26,27] have indicated the effectiveness of GFRP shear friction reinforcement in enhancing the interface shear capacity of joints with varying surface roughness. Additionally, specimens reinforced with GFRP exhibited notable post-peak residual capacity.

Given the expanding use of GFRP bars in structural applications, there is a clear need for experimental evidence that clarifies how monolithically uncracked concrete interfaces behave when reinforced with GFRP. Unlike pre-cracked or cold-jointed concrete interfaces, initially uncracked monolithic interfaces do not contain a pre-existing discontinuity along the potential shear plane. Consequently, their shear-transfer behavior involves the initiation and propagation of a new interface crack before the interface develops the mechanisms typically associated with an established crack or construction joint, such as aggregate interlock and friction. The presence of transverse reinforcement further influences this process through clamping and dowel action, particularly after cracking. In fact, the transfer of shear forces across monolithic, uncracked interfaces is a common requirement in various structural configurations. Typical examples include the interface between a column and an integrated corbel, as seen in Figure 2a, or the junction connecting the thin web to the wide bottom flange in prestressed I-girders, as shown in Figure 2b. In this context, the present study experimentally investigates the shear transfer behavior of initially uncracked concrete interfaces reinforced with GFRP clamping reinforcement. The influence of key parameters, including reinforcement type and ratio, on cracking, ultimate, and residual shear strengths is examined. Additionally, the experimental findings are compared with predictions from existing models to evaluate their accuracy. The results of this study are expected to contribute to a better understanding of shear transfer mechanisms in GFRP-reinforced systems and to support the development of improved design recommendations.

## 2. Experimental Program

### 2.1. Push-Off Specimens

The experimental program involved the fabrication and testing of twelve push-off specimens, which were loaded until failure. The specimens were cast monolithically using the same concrete batch and remained uncracked prior to the application of shear loading. All specimens were identical in size, with dimensions of 200 mm in thickness, 400 mm in width, and 800 mm in height as shown in Figure 3. The two L-shaped parts were separated by a 25 mm gap, allowing for measurable deformation and enabling the characterization of post-peak behavior under loading. A 20 mm-deep notch was provided on both faces of the specimen to promote crack initiation along the interface plane. This configuration produced a shear plane area measuring 160 mm by 350 mm. The test variables included the type and amount of clamping reinforcement crossing the interface plane. The details of the test matrix are presented in Table 1. The table indicates that eight specimens were reinforced with GFRP clamping stirrups with reinforcement ratio *ρ_v_* ranging from 0.28 to 1.9%. Four of the GFRP specimens were reinforced with 10 mm–diameter stirrups with *ρ_v_* ranging from 0.28 to 1.12%. These ratios were achieved by increasing the number of stirrups from one two-legged stirrup at the lowest *ρ_v_* to four stirrups at the highest *ρ_v_* for this bar size. The remaining four GFRP specimens were reinforced with 13 mm–diameter stirrups, using a similar number of stirrups to their counterparts with 10 mm–diameter bars, resulting in *ρ_v_* ranging from 0.47% to 1.9%. The test matrix also comprised three specimens reinforced with 10 mm–diameter steel clamping stirrups with *ρ_v_* ranging from 0.28 to 0.84%. An additional specimen without stirrups crossing the interface plane was included to isolate and evaluate the contribution of concrete to the shear resistance. Each specimen was reinforced within the L-shaped segments using longitudinal and transverse steel reinforcement to prevent premature failure caused by concrete crushing or splitting under the applied loading, as illustrated in Figure 3. The designation of the specimen reflects its key characteristics. The letter, “S” or “G”, indicates the type of clamping reinforcement: steel or GFRP, respectively. The first number represents the number of stirrup legs crossing the interface plane, while the second number denotes the stirrup diameter. The control specimen without interface stirrups is designated as “C”. Key stages of the assembly and casting process are documented in Figure 4.

### 2.2. Material Properties

Ready-mix normal-weight concrete from a local plant was used to cast the specimens, with a specified compressive strength of 40 MPa at 28 days. The actual concrete compressive strength was determined on the day of testing by conducting compression tests on standard 150 × 300 mm cylinders following ASTM C39/C39M-23 [28]. The average compressive strength, obtained from three cylinders, was 46.8 MPa, with a standard deviation of 0.4 MPa.

GFRP U-shaped rebars, as illustrated in Figure 5, were used as clamping reinforcement for the interface plane. Two rebars were assembled to form a single two-legged stirrup crossing the interface plane as shown in Figure 3. All GFRP rebars were provided from the same manufacturer and were characterized by helically wrapped surface profile to enhance bonding with concrete. Two GFRP bar sizes, No. 10 and No. 13, were considered in this study. The mechanical properties of the bars, as provided by the manufacturer, are summarized in Table 2. Deformed steel bars of 10 mm diameter were used as clamping reinforcement for the interface plane for some specimens. The additional steel used for reinforcing the L-shaped parts included 12 mm diameter longitudinal bars and 8 mm diameter stirrups. The mechanical properties of the steel bars were determined in accordance with ASTM A370-19e1 [29] and are presented in Table 2.

### 2.3. Test Setup and Instrumentation

Figure 6a shows schematically the configuration of the push-off test setup. The specimens were positioned vertically and subjected to concentric loading using AMSLER testing machine (Alfred J. Amsler & Co., Schaffhausen, Switzerland). This configuration was adopted to generate pure shear along the interface plane without introducing any associated bending moment. The load was applied in a monotonic manner using displacement control at a rate of 0.1 mm/min. To monitor the strain during the loading process, electrical resistance strain gauges were bonded to the clamping reinforcement at the interface. A vertical LVDT was mounted on each face of the specimen to record the shear slip between the L-shaped parts along the interface plane. Crack opening was monitored using a pair of small LVDTs installed horizontally on each face of the specimen. All instruments were wired to a data acquisition system to ensure continuous recording of the experimental measurements. Figure 6b illustrates the test setup and external instrumentation.

## 3. Experimental Results

The key experimental results obtained from the testing program are summarized in Table 3. The shear stress was determined by dividing the applied load by the nominal area of the shear friction transfer plane, which measured 160 mm by 350 mm. Detailed discussions regarding the main experimental findings are provided in the following sections.

### 3.1. Deformation Response and Mode of Failure

Figure 7 plots the relationship between the shear stress and the shear deformation (vertical slip) along the transfer plane. For clarity, the results are grouped according to the type and size of the clamping reinforcement. Figure 7a presents the response of the specimens reinforced with steel stirrups, whereas Figure 7b,c correspond to the specimens with GFRP stirrups of 10 mm– and 13 mm–diameter, respectively. The response of the control specimen is shown in Figure 7d.

As illustrated in Figure 7, the shear stress–slip response of all specimens with clamping reinforcement can generally be divided into three distinct parts. The first part, extending from starting the load up to the cracking of specimens, exhibits a linear response and is approximately similar to all specimens irrespective of the type and amount of clamping reinforcement. This part is characterized by negligible slip under loading, with the recorded slip values at the onset of cracking (*v_cr_*) being approximately 0.01 mm for all specimens tested in this study. This suggests that the shear resistance during this stage is primarily governed by the cohesion of the concrete.

The second part of the shear stress–slip curve extends from the onset of cracking (*v_cr_*) to the ultimate shear resistance (*v_u_*). Following crack initiation, slip develops at a noticeably faster rate than observed in the first part of the curve. Within this part, the behavior seems to be influenced by both the type and amount of clamping reinforcement. Due to the higher modulus of elasticity of steel reinforcement, specimens reinforced with steel clamps experienced lower slip values compared with their counterparts reinforced with the same amount of GFRP. For example, specimen S–6–10 recorded a slip of 0.03 mm compared to 0.17 mm for specimen G–6–10 at a shear stress of 6 MPa. Increasing the amount of clamping reinforcement for the same material type of reinforcement resulted in a stiffer behavior, characterized by lower slip values at the same level of shear stress. For all specimens with clamping reinforcement tested in this study, the lowest slip value at *v_u_* was 0.24 mm as shown in Table 3, recorded for specimen S–2–10, which had the lowest ratio of steel clamping reinforcement. In contrast, Table 3 indicates that specimen G–2–10, which contained the minimum quantity of GFRP reinforcement, exhibited the largest slip value of 0.77 mm at *v_u_*. The consistently low slip values recorded for all specimens prior to reaching *v_u_* indicate that only limited relative displacement occurred along the crack interface and that no significant localized deformation developed in the clamping bars. Consequently, the clamping bars were not subjected to sufficient transverse deformation to effectively mobilize dowel action, rendering its contribution to the overall shear transfer mechanism negligible. Instead, the shear resistance during this stage was primarily governed by the shear friction mechanism activated along the crack interface as a result of the clamping action provided by the transverse reinforcement, in addition to the residual concrete cohesion that remained active across the cracked surface. The combination of these mechanisms enabled the specimens to sustain increasing levels of shear stress while maintaining relatively small slip values up to the ultimate shear resistance, *v_u_*.

The third part of the shear stress–slip curve is represented by the descending branch of the curve up to the end of testing. During this post-peak phase, the interface undergoes structural softening characterized by a substantial increase in slip displacement, resulting in a gradual deterioration of the interfacial integrity and the eventual separation of the two crack planes. This was accompanied by a sharp drop in shear stress which can be attributed to the deterioration of concrete cohesion and the gradual loss of shear friction as slip increases. Nevertheless, the presence of clamping reinforcement enabled the specimens to maintain a residual level of shear resistance, *v_r_*, during the post-peak response, which was mainly attributed to the dowel action of the clamping reinforcement. As illustrated in Figure 7d, the control specimen without clamping reinforcement exhibited excessive slip accompanied by a complete loss of interfacial shear resistance and structural integrity immediately upon reaching the cracking capacity.

Figure 8 depicts the evolution of the horizontal crack width along the transfer plane with increasing shear stress, highlighting the cracking behavior of the tested specimens. To maintain consistency, the graphs in this figure are organized into groups matching the layout of Figure 7. It can be observed from Figure 8 that the shear stress-crack width curves for specimens with clamping reinforcement are consisted of three main parts: pre-carking, post-cracking up to peak stress, and post-peak softening. This response closely mirrors the behavioral trends established for the shear stress–slip relationships in Figure 7. Additionally, it is apparent that the crack opening is also affected by the amount and type of clamping reinforcement utilized. The crack width recorded at the initiation of cracking ranged from 0.04 to 0.06 mm for the tested specimens. On the other hand, the recorded values at *v_u_* ranged between 0.68 and 2.15 mm as presented in Table 3. The lowest value was observed for specimen S–6–10, whereas the highest value was recorded for specimen G–2–10, confirming the trends observed in the corresponding slip measurements.

Experimental observations indicated that all test specimens exhibited a similar mode of failure that occurred along the plane of the shear interface. This mode of failure was characterized by the formation of vertical crack along the interface plane. In the absence of clamping reinforcement, the control specimen exhibited highly brittle behavior. Once the vertical crack initiated, it propagated instantaneously across the interface plane, shearing off the aggregate and causing abrupt splitting of the member and separation of the L-blocks. On the other hand, specimens provided with either steel or GFRP clamping reinforcement did not exhibit such brittle and sudden failure. Following the initiation of the vertical crack, the clamping reinforcement was activated, developing tensile stresses that restricted crack opening through the shear friction mechanism. This internal clamping force maintained aggregate interlock along the shear plane, allowing the specimens to sustain substantial shear resistance and exhibit notable post-peak residual capacity. After failure, the clamping stirrups remained intact in all reinforced specimens, with no evidence of rupture. Figure 9 illustrates the typical mode of failure observed for each group of test specimens along with the control specimen. In some cases, concrete spalling was observed near the boundaries of the interface plane, specifically at the intersection where the shear interface meets the termination edge of the gap.

### 3.2. Strain in Clamping Stirrups

The relationship between the applied shear stress and the strain developed in the clamping stirrups is presented in Figure 10 for the tested specimens. The figure clearly demonstrates that the strain developed in the stirrups during the uncracked stage was minimal. This indicates that the stirrups were not actively engaged during this stage, and their contribution to the initial shear transfer was negligible. Consequently, the shear transfer mechanism at this stage was primarily governed by the concrete cohesion across the interface. After cracking, a noticeable increase in the stirrup strain was observed as the clamping reinforcement became engaged in resisting the applied shear force. The increase in strain reflects the development of tensile stresses in the stirrups, which provided confinement across the shear interface. This confinement sustained the aggregate interlock mechanism, allowing continuous mobilization of the shear-friction resistance. As the applied stress approached the peak value, the stirrup strain continued to increase, indicating their significant contribution to maintaining shear resistance and delaying failure. Thus, the overall shear transfer mechanism during this stage of post-cracking shifts from being dominated by concrete action, represented by cohesion, to a state primarily governed by shear friction along the interface, with a contribution from the residual concrete cohesion. The post-peak behavior of the test specimens shown in Figure 10 was characterized by the drop in shear resistance accompanied by the increase in strain developed in the stirrups. At this stage, the aggregate interlock mechanism and concrete cohesion were progressively degraded, resulting in a reduction in the shear resistance to the residual level. The residual shear capacity was primarily sustained by the dowel action provided by the clamping stirrups.

Figure 10 demonstrates that the strain variation during the post-cracking and post-peak stages is influenced by the type and quantity of clamping stirrups. By comparing the strain behavior of specimens reinforced with steel stirrups in Figure 10a with their counterparts reinforced with GFRP stirrups of the same size and amount in Figure 10b, one can observe that the steel-reinforced specimens showed stiffer behavior in the post-cracking stage. Additionally, the reduction in shear resistance from the peak to the residual level occurred more gradually in the steel-reinforced specimens compared with the GFRP-reinforced specimens. This can be attributed to the higher modulus of elasticity of the steel stirrups, which results in a greater reinforcement stiffness (*ρ_v_E*) compared with that provided by GFRP stirrups, as presented in Table 1. Furthermore, Figure 10 shows that, for the same type and size of reinforcing clamps, increasing *ρ_v_* resulted in a stiffer strain response during the post-cracking stage. This effect was more evident in the steel-reinforced specimens shown in Figure 10a and in the GFRP-reinforced specimens with 13 mm–diameter stirrups shown in Figure 10c. Also, increasing *ρ_v_* by increasing the stirrup diameter from 10 mm to 13 mm resulted in a similar response, as demonstrated by comparing Figure 10c with Figure 10b.

Table 3 lists the strain values of the clamping stirrups measured at *v_u_*. The values of the stirrup strain in the steel-reinforced specimens ranged between 2420 and 3250 με. For this strain range, only the steel stirrups in specimen S–2–10 reached strain values exceeding the yield strain of 2925 με for the steel bars used in this study. The strains developed in stirrups of specimens S–4–10 and S–6–10 were 83% and 91% of the yield strain, respectively. Due to the relatively lower modulus of elasticity of the GFRP reinforcement, the strain values measured in the GFRP stirrups at *v_u_* were higher than those measured in the steel stirrups. For specimens reinforced with 10 mm–diameter GFRP stirrups, the measured strain values ranged from 5036 to 9820 µε, with the maximum strain recorded in specimen G–2–10, as given in Table 3. This measured strain range represents approximately 23–45% of the ultimate strain capacity of the 10 mm–diameter GFRP stirrups. The corresponding range for specimens reinforced with 13 mm–diameter GFRP stirrups, was 4120 to 6522 µε, representing approximately 21–43% of the ultimate strain capacity of the stirrups. The obtained results indicate that the average strain developed in the GFRP stirrups at the ultimate shear resistance of the test specimens was approximately twice that measured in the steel stirrups.

### 3.3. Cracking Shear Resistance

The cracking shear stress, *v_c__r_*, corresponding to the initiation of the first visible surface crack, is given in Table 3. For all tested specimens, the onset of cracking was characterized by the formation of a vertical crack that extended along the shear interface plane. Figure 11 presents the relationship between the cracking shear stress and the axial stiffness parameter, *ρ_v_E*, of the clamping stirrups for the tested concrete interfaces. In general, the experimental results indicate that the cracking shear stress remains relatively constant, fluctuating tightly between 3.84 and 4.6 MPa across a wide range of *ρ_v_E* values (spanning from 140 to 1684 MPa). Notably, no clear increasing or decreasing trend in cracking resistance was observed with increasing reinforcement ratio or elastic modulus. This consistent behavior was observed across all three groups of tested specimens. The slight scatter in the results, particularly for the steel-reinforced specimens and the 13 mm–diameter GFRP-reinforced specimens, falls within the expected range of variability associated with experimental testing and does not indicate a significant influence of the clamping reinforcement stiffness on the cracking shear stress. The obtained cracking shear stress values were also comparable to that of the control specimen without clamping stirrups, which exhibited a cracking shear stress of 4.29 MPa as given in Table 3. These findings demonstrate that the initial cracking stress of the interface of monolithic specimens of this study is predominantly governed by the cohesive tensile strength of the concrete matrix itself, rather than the mechanical properties, diameter, or stiffness of the intersecting reinforcement crossing the interface plane.

### 3.4. Ultimate Shear Resistance

Table 3 presents the measured ultimate shear strength, *v_u_*, of the tested concrete interfaces. The ultimate shear strength was determined based on the peak load recorded during testing and represents the maximum shear stress developed across the interface before failure. Also, Figure 12 presents the relationship between the ultimate shear strength and the reinforcement stiffness parameter *ρ_v_E*. Unlike the cracking shear strength, which remained independent of the clamping reinforcement characteristics, the ultimate shear strength showed a clear increasing trend with increasing *ρ_v_E*. This improvement was observed for both steel- and GFRP-reinforced specimens, indicating that the increased confinement provided by the tensile force developed in the clamping reinforcement contributed to maintaining shear transfer mechanisms across the interface after cracking.

Within each specimen group having the same stirrup diameter, the change in *ρ_v_E* resulted from the variation in the clamping reinforcement ratio, *ρ_v_*. This was accomplished by increasing the amount of transverse reinforcement, where the number of stirrups was increased from a single two-leg stirrup to two or three stirrups for steel-reinforced specimens and to two, three, or four stirrups for GFRP-reinforced specimens. For each group of specimens, it can be seen that increasing *ρ_v_* resulted in an increase in *v_u_*. Increasing *ρ_v_* from 0.28% to 0.56% resulted in increases in *v_u_* of 50% and 22% for the steel- and GFRP-reinforced specimens with 10 mm-diameter stirrups, respectively. A further increase in *ρ_v_* from 0.28% to 0.84% led to corresponding increases in *v_u_* of 67% and 36%, respectively. An increase of 53% in *v_u_* was obtained when *ρ_v_* of GFRP reinforcement was increased from 0.28% to 1.12%. For the test specimens reinforced with 13 mm-diameter clamps, increasing *ρ_v_* from 0.47% to 0.95% did not show any noticeable increase in *v_u_* where both specimens provided approximately the same capacity. However, further increases in *ρ_v_* to 1.4% and 1.9, led to significant improvements in *v_u_*, with increases of 22% or 44%, respectively.

Each group of GFRP-reinforced specimens included corresponding specimens with identical numbers and spacing of GFRP stirrups as those in the other group. The only difference between each pair of corresponding specimens was *ρ_v_*, which was increased by increasing the stirrup diameter from 10 mm to 13 mm while maintaining the same stirrup configuration. A comparison of the results obtained for the two groups, considering the variation in *ρ_v_* within the specimens of the group, indicates that the influence of *ρ_v_* was more pronounced in the specimens reinforced with 10 mm-diameter GFRP stirrups. For this group, increasing *ρ_v_* resulted in a consistent increase in *v_u_* over the entire range of reinforcement ratios investigated, as shown in Figure 12 and indicated previously. On the other hand, a comparison between the obtained results for any two corresponding specimen pairs from the two groups did not reveal a consistent trend. Increasing *ρ_v_* from 0.28% (specimen G–2–10) to 0.47% (specimen G–2–13) or from 1.12% (specimen G–8–10) to 1.9% (specimen G–8–13) increased *v_u_* by 12% and 5%, respectively. However, increasing *ρ_v_* from 0.56% (specimen G–4–10) to 0.95% (specimen G–4–13) or from 0.84% (specimen G–6–10) to 1.4% (specimen G–6–13) did not result in any increase in *v_u_*, as shown in Figure 12 and summarized in Table 3. These findings suggest that simply increasing *ρ_v_* by using larger-diameter stirrups does not necessarily lead to a proportional increase in shear resistance. The effectiveness of the larger stirrups appeared to depend on the reinforcement level, with improvements observed only at the lowest and highest reinforcement ratios investigated in this study. Even these improvements (5% to 12%) were relatively small considering the substantial increase in *ρ_v_* achieved by using larger-diameter stirrups. This behavior may be attributed to the anisotropic nature of GFRP bars and the nonuniform utilization of their cross-sectional fibers during interface opening. Although the larger-diameter bars provide a greater nominal reinforcement area, the increase in effective tensile contribution is not necessarily proportional to the increase in area. In addition, the larger bar diameter may alter the local interaction between the GFRP bars and the surrounding concrete, resulting in localized stress concentrations near the interface. Consequently, the additional reinforcement area provided by the 13 mm bars may not be fully effective in mobilizing additional shear-transfer resistance, leading to an inconsistent increase in ultimate shear strength.

A comparison was also made between the obtained results of GFRP-reinforced specimens with 10 mm-diameter stirrups and their steel-reinforced counterparts. The steel-reinforced group included three specimens with the same stirrup diameter, *ρ_v_*, and stirrup spacing as the corresponding GFRP-reinforced specimens. Therefore, the main variable between the two groups was the reinforcement stiffness *ρ_v_E* which varied due to the higher modulus of elasticity *E* of the steel reinforcement compared with that of the GFRP reinforcement. Considering the variation in *ρ_v_* within the specimens of each group, the steel-reinforced specimens showed larger enhancement (50% to 67%) in *v_u_* compared with their counterparts of GFRP-reinforced specimens (22% to 36%), as indicated previously. Also, a direct comparison between each corresponding pair of steel- and GFRP-reinforced specimens showed that specimens S–2–10, S–4–10, and S–6–10 exhibited increases in *v_u_* of 15%, 42%, and 42%, respectively reference to their counterparts G–2–10, G–4–10, and G–6–10. This is attributed to the higher modulus of elasticity of steel, which provided greater clamping stiffness across the shear interface. Consequently, the steel stirrups were more effective in enhancing the shear-friction mechanism by restraining the opening of the shear crack, thereby maintaining aggregate interlock and resulting in higher *v_u_*.

The obtained results and discussion strongly indicate that while the reinforcement stiffness parameter *ρ_v_E* is an influential parameter in predicting ultimate interface shear capacity, it cannot be used as a standalone indicator. This fact is further supported by comparing specimens S–2–10 and G–8–10, as shown in Figure 12 and Table 3. Although specimen S–2–10, reinforced with a single two-leg steel stirrup, had the same reinforcement stiffness parameter *ρ_v_E* of 561 MPa as specimen G–8–10, reinforced with four-leg GFRP stirrups, the two specimens exhibited markedly different values of *v_u_*. Specimen G–8–10 achieved a *v_u_* of 8.73 MPa, compared with only 6.55 MPa for specimen S–2–10. These results demonstrate that, although *ρ_v_E* is an important parameter governing the interface shear capacity, it does not fully characterize the shear behavior. The distribution and spacing of the clamping stirrups also play a significant role, as a more uniformly distributed reinforcement provides more effective crack restraint and aggregate interlock along the shear interface, thereby enhancing the ultimate shear resistance.

Table 3 further demonstrates the beneficial effect of clamping reinforcement, as all reinforced specimens achieved higher values of *v_u_* than the control specimen (C) without clamping reinforcement. For the steel-reinforced group, the increase in *v_u_* ranged from 53% to 155% relative to the control specimen. The corresponding increases in *v_u_* for the GFRP-reinforced specimens with 10 mm– and 13 mm–diameter stirrups ranged from 33% to 103% and from 46% to 113%, respectively. It should be pointed out that the CSA S6 code [15] recommends minimum values of 0.45% and 225 MPa for *ρ_v_* and *ρ_v_E*, respectively, for GFRP-reinforced interfaces. However, the lowest reinforcement level investigated in this study, represented by specimen G–2–10, had a *ρ_v_* of only 0.28% and a corresponding *ρ_v_E* of 140 MPa; both of which were below the minimum values specified by the CSA S6 code. Nevertheless, specimen G–2–10 developed a reserve strength of 33% above that of the unreinforced control specimen.

### 3.5. Residual Shear Resistance

The post-peak residual shear resistance, *v_r_*, is summarized in Table 3 for each specimen and Figure 13 illustrates the relationship between *v_r_* and *ρ_v_E* for the steel-reinforced specimens and the two groups of GFRP-reinforced specimens with 10 mm– and 13 mm–diameter stirrups. In general, the residual shear resistance increased with increasing *ρ_v_E* for all three groups, demonstrating that the clamping reinforcement remained effective after the peak load by providing dowel action across the shear interface and preventing complete separation of the two concrete segments.

A consistent trend was observed for all specimen groups, with *v_r_* increasing as *ρ_v_* increased. This improvement can be attributed to the greater number of stirrup legs crossing the shear interface at higher reinforcement ratios, which enhanced the dowel action of the clamping reinforcement and, consequently, the residual shear resistance. Specimens S–2–10, S–4–10, and S–6–10 reinforced with steel stirrups exhibited *v_r_* of 2.73 MPa, 4.44 MPa, and 7.41 MPa, respectively. This indicates that increasing *ρ_v_* from 0.28% to 0.56% and from 0.28% to 0.84% resulted in increases in *v_r_* of 63% and 171%, respectively. The corresponding specimens with GFRP stirrups, G–2–10, G–4–10, and G–6–10, experienced *v_r_* of 3.17 MPa, 3.7 MPa, and 5.01 MPa, respectively. Thus, increasing *ρ_v_* from 0.28% to 0.56% and from 0.28% to 0.84% resulted in increases in *v_r_* of 17% and 58%, respectively. Although specimen G–2–10 exhibited a slightly higher residual shear resistance than its steel-reinforced counterpart S–2–10, the steel-reinforced specimens outperformed the corresponding GFRP-reinforced specimens at higher reinforcement ratios. In particular, specimens S–4–10 and S–6–10 exhibited residual shear resistances that were approximately 20% and 48% higher, respectively, than those of specimens G–4–10 and G–6–10. This comparison indicates that the higher stiffness of the steel stirrups became increasingly effective in sustaining the post-peak shear transfer mechanisms as the clamping reinforcement ratio increased, resulting in a more pronounced enhancement in residual shear resistance than that achieved with GFRP reinforcement. Further increasing *ρ_v_* of the GFRP reinforcement from 0.28% to 1.12% resulted in a 102% increase in *v_r_*. Specimens G–2–13, G–4–13, G–6–13, and G–8–13 exhibited *v_r_* of 2.78 MPa, 4.24 MPa, 5.53 MPa, and 7.23 MPa, respectively. Reference to *v_r_* of specimen G–2–13, these results represent increases in *v_r_* of 52%, 99%, and 160%, respectively.

The results presented in Figure 13, together with the preceding discussion, further confirm the conclusion reached for the peak shear resistance *v_u_* that the reinforcement stiffness parameter *ρ_v_E* alone is insufficient to accurately characterize the residual shear resistance *v_r_* in this case. In addition to reinforcement stiffness, the distribution and spacing of the clamping stirrups play a critical role in mobilizing the dowel action mechanism. A more uniform distribution of stirrups along the shear interface provides more effective dowel action, resulting in improved post-peak shear transfer and higher residual shear resistance.

Table 3 also presents the ratio of the residual shear resistance to the peak shear resistance *v_r_*/*v_u_* for each reinforced specimen. The steel-reinforced specimens exhibited *v_r_*/*v_u_* ratios ranging from 0.42 to 0.68. On the other hand, the GFRP-reinforced specimens with 10 mm–diameter stirrups showed ratios between 0.53 and 0.73, while those reinforced with 13 mm–diameter stirrups exhibited ratios ranging from 0.44 to 0.79. The generally higher *v_r_*/*v_u_* ratios of the GFRP-reinforced specimens indicate that, although they developed lower peak shear resistance than the steel-reinforced specimens, they retained a larger proportion of their peak shear capacity during the post-peak stage.

## 4. Comparison of Experimental Results and Analytical Predictions

In this section, the experimentally determined ultimate shear resistance of the GFRP-reinforced specimens is compared with the predictions obtained using three available shear-friction models. These models comprise two code-based formulations, namely the AASHTO LRFD model [30] and CSA S6-25 model [15], and one research-based model proposed by Vega et al. [31]. It should be noted that ACI 440.11-22 code [17] does not provide specific provisions for the design of shear-friction interfaces.

### 4.1. AASHTO LFRD Model

The shear friction provisions specified in the AASHTO LFRD bridge design guide specifications for GFRP reinforced concrete [30] are derived from those in the conventional AASHTO LFRD bridge design specifications [22], which are based on the cohesion-plus-friction concept. The interface resistance is assumed to consist of a cohesion component and a friction component arising from the normal force developed in the reinforcement crossing the interface and any permanent external compressive force. In the GFRP adaptation, the design tensile strength, *f_fd_*, of GFRP reinforcement is used for calculating the friction component of the shear resistance. The model is expressed as follows:

(1) $$v_{AASHTO}=c+\mu \left(\rho_{v}f_{fd}+\frac{P_{c}}{A_{cv}}\right)\leq \left[\begin{array}{c} 0.25f_{c}^{\prime }\\ 10.3\;MPa \end{array}\right]$$

where *c* and *µ* are the concrete cohesion and coefficient of friction, respectively, and are taken as 2.76 MPa and 1.4 for monolithic construction; *ρ_v_* is the ratio of the interface shear reinforcement crossing the shear plane; *P_c_* is the permanent compressive force acting normal to the shear plane; *A_cv_* denotes the area of the concrete interface; and $f_{c}^{\prime }$ stands for the specified compressive strength of concrete.

### 4.2. CSA S6 Model

The CSA S6-25 code [15] adopts a different approach for accounting for GFRP interface reinforcement. Rather than assuming that the GFRP reinforcement reaches its full tensile capacity at interface failure, the GFRP contribution is expressed in terms of an equivalent stress corresponding to a specified strain limit. The model is given by the following equations for calculating the design shear resistance:

(2) $$v_{CSA}=\emptyset_{c}\left[c+\mu \left(\rho_{v}E_{f}\varepsilon_{f}+\frac{P_{c}}{A_{cv}}\right)\right]\leq 0.25\emptyset_{c}f_{c}^{\prime }$$

(3) $$\rho_{v}E_{f}\geq 225\;MPa$$

(4) $$\rho_{v}\geq 0.45\%$$

where *c* and *µ* are taken as 1 MPa and 1.4, respectively, for monolithically cast concrete; *ϕ_c_* is the concrete reduction factor; and *E_f_* and *ε_f_* are the GFRP modulus of elasticity and strain limit, respectively, with *ε_f_* specified by the code as 0.004.

### 4.3. Vega et al. Model

To overcome the limitations of applying steel-based shear-friction provisions directly to GFRP reinforcement, Vega et al. [31] proposed a regression-based model specifically tailored to GFRP-reinforced interfaces. In their proposed model, they retained AASHTO’s cohesion-plus-friction model structure, but instead of using the full tensile strength of the GFRP, they introduced a stress limit governed by a maximum GFRP strain ($\varepsilon_{f}$) of 0.002.

(5) $$v_{Vega}=c+\mu \left(\rho_{v}E_{f}\varepsilon_{f}+\frac{P_{c}}{A_{cv}}\right)\leq \left[\begin{array}{c} \begin{array}{c} 0.2f_{c}^{\prime }\\ 3.3+0.08f_{c}^{\prime } \end{array}\\ 11\;MPa \end{array}\right]$$

The model adopts the same cohesion and friction coefficients specified by AASHTO [28] for monolithic construction, with values of 2.76 MPa and 1.4, respectively. Also, they adopted the upper limit for interface shear resistance specified by ACI 318 [32].

### 4.4. Experimental Versus Predicted Ultimate Shear Strength

To assess the accuracy of the three analytical models, their ultimate shear strength predictions for the GFRP-reinforced specimens were compared with the corresponding experimental results, as summarized in Table 4 and illustrated in Figure 14. It should be pointed out that the concrete reduction factor *ϕ_c_* in Equation (2) was taken as unity for the analytical calculations. Furthermore, the value of *Pc* was set to zero in Equations (1), (2) and (5), as no permanent compressive force was applied to the specimens during testing. The value of *f_fd_* in Equation (1) was taken as the ultimate tensile strength of the GFRP bars reported in Table 2, without applying an environmental reduction factor.

The comparison presented in Table 4 and Figure 14 indicates noticeable differences in the accuracy and conservatism of the three prediction methods. The AASHTO [30] formulation generally overestimates the experimental interface shear strength (*v_u_*), as reflected by ratios of *v_u/_v_AASHTO_* below unity for all specimens. The average ratio *v_u/_v_AASHTO_* of 0.75 indicates that the AASHTO [30] model predicts, on average, interface shear strengths approximately 33% higher than those obtained experimentally. Therefore, its predictions are generally nonconservative for the tested GFRP-reinforced interfaces. This outcome is expected because the AASHTO [30] formulation employs the design tensile strength of GFRP (*f_fd_*) in Equation (1), which is substantially higher than the yield strength of conventional steel for which the original equation was developed.

In contrast, the CSA S6 [15] provisions generally provide unduly conservative predictions, with ratios greater than unity for all specimens. The average ratio *v_u/_v_CSA_* of 2.19 indicates that the experimental interface shear strengths are, on average, more than twice the values predicted by the CSA S6 [15] provisions. This demonstrates a substantial degree of conservatism, although the relatively high CoV of 29.2% indicates considerable scatter and limited consistency among the predictions. As shown in Figure 14b, the degree of conservatism increases as the reinforcement ratio or the axial stiffness of the clamping reinforcement decreases. It should be noted that specimen G-2-10, which had the lowest reinforcement ratio (0.28%) and axial stiffness (140 MPa) among the specimens tested, both below the minimum values specified by Equations (3) and (4), exhibited the most conservative *v_u/_v_CSA_* ratio of 3.19. These overly conservative and highly scattered predictions obtained using the CSA S6 [15] method may be attributed to the relatively low concrete cohesion value, *c* = 1.0 MPa, specified for monolithic construction.

The Vega et al. [31] model also provides conservative predictions, with *v_u/_v_Vega_* ratios ranging from 1.52 to 2.01 and an average value of 1.79. Thus, the experimental interface shear strengths are, on average, approximately 79% higher than the predicted values. Although the model is more conservative than the AASHTO formulation, it exhibits the lowest CoV of 10.4%, indicating the highest level of consistency among the three methods. Moreover, its predictions are less conservative than those of the CSA S6 [15] method, as indicated by the lower average ratio of *v_u/_v_Vega_*, despite using a lower GFRP strain ($\varepsilon_{f}$) limit than the value of 0.004 specified by CSA S6 [15] method. This difference can be attributed to the relatively higher concrete cohesion value, *c* = 2.76 MPa, adopted by Vega et al. [31], compared with the lower value of 1.0 MPa specified by CSA S6 [15] method for monolithic construction.

## 5. Conclusions

The shear transfer behavior of initially uncracked concrete interfaces reinforced with GFRP clamping stirrups was experimentally investigated through twelve push-off specimens subjected to monotonic loading. The experimental results were also compared with predictions from available shear-friction models. The main conclusions are as follows:

1. Increasing the clamping reinforcement ratio enhanced both the ultimate and residual interface shear strengths, while the cracking shear strength was essentially unaffected by the clamping reinforcement characteristics.
2. The specimens reinforced with GFRP clamping reinforcement provided lower ultimate interface shear strength than those reinforced with steel reinforcement, but exhibited more favorable post-peak behavior by retaining a greater proportion of the peak resistance. None of the reinforced specimens experienced rupture of the clamping reinforcement.
3. The interface shear response was governed not only by the stiffness and amount of clamping reinforcement but also by its distribution and bar diameter. Therefore, reinforcement ratio or axial stiffness alone may not be sufficient to fully characterize the shear-transfer behavior of reinforced concrete interfaces.
4. The predictions of the AASHTO LRFD, CSA S6, and Vega et al. models showed different levels of agreement with the experimental results. The AASHTO LRFD method provided unconservative predictions, whereas CSA S6 and Vega et al. yielded conservative predictions. Among the three approaches, the Vega et al. model provided the best overall balance of accuracy, conservatism, and consistency, although its conservatism could potentially be reduced for closer agreement with the experimental results.

## Figures and Tables

**Figure 1:**
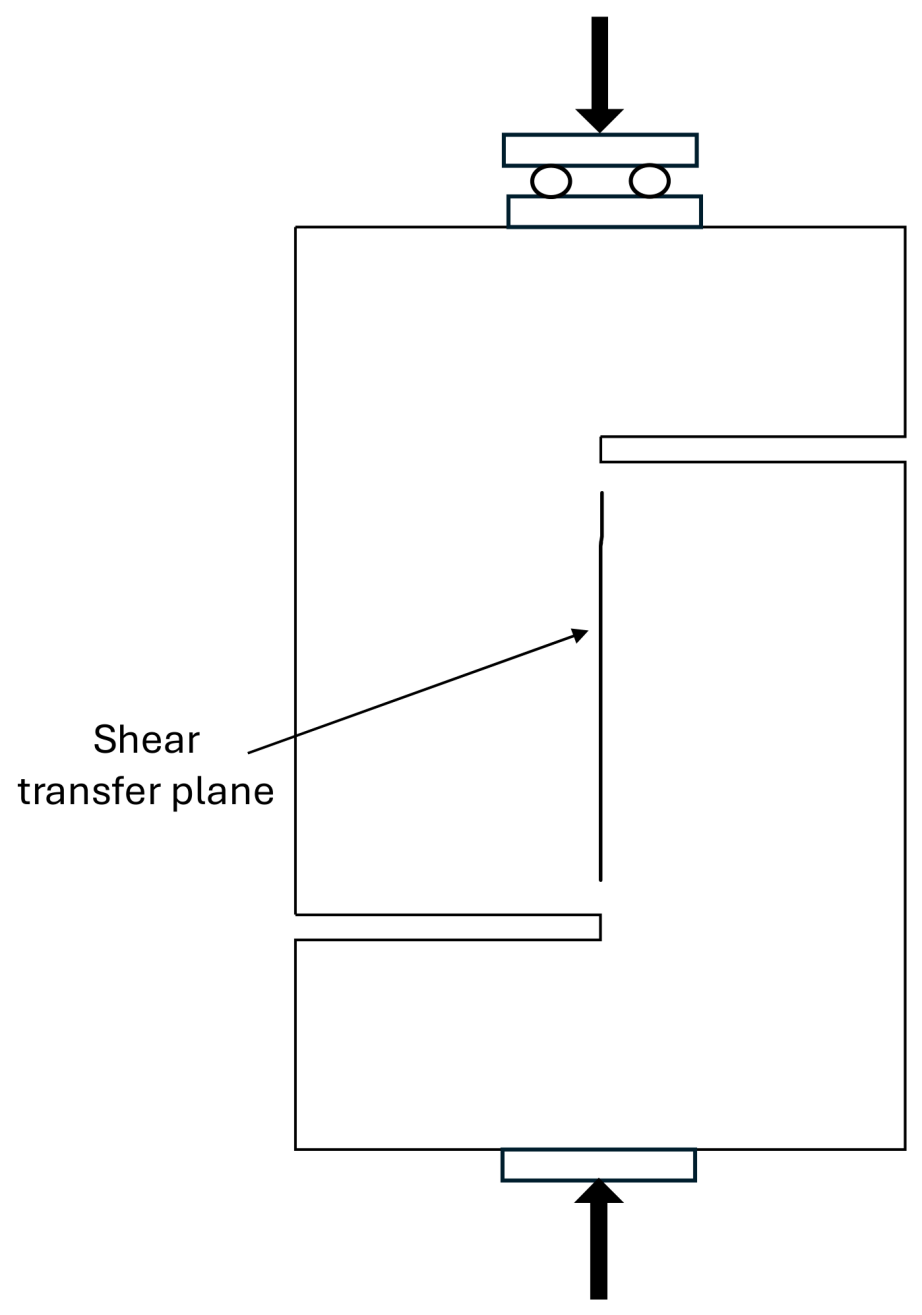
Typical push-off test specimen.

**Figure 2:**
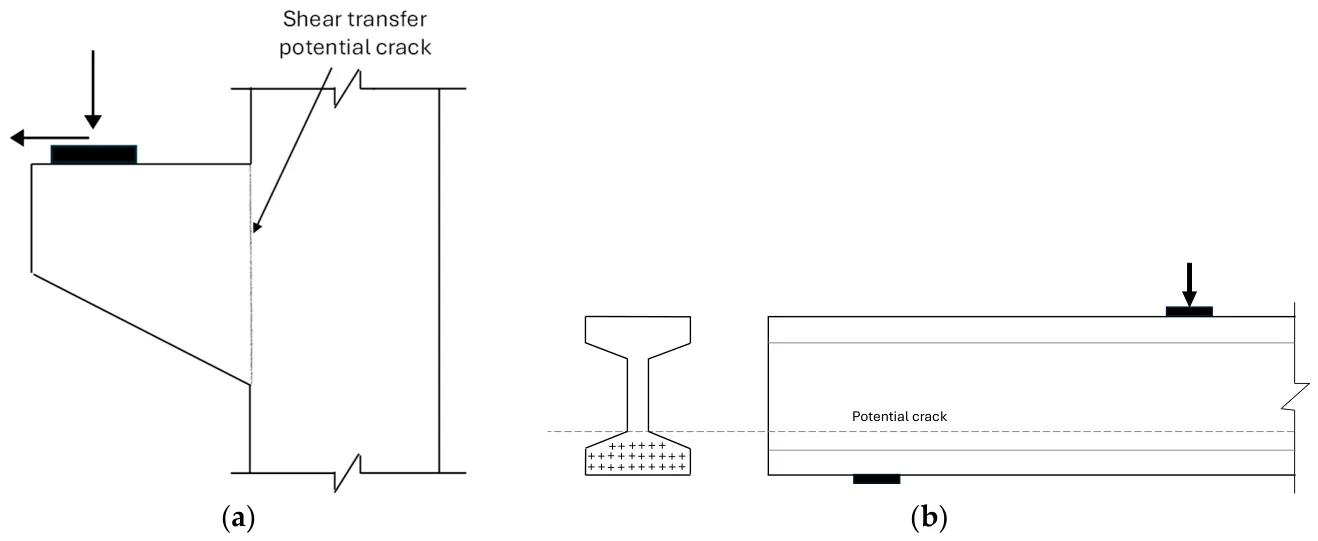
Shear transfer across initially uncracked concrete planes: (**a**) Corbel; (**b**) Prestressed I-beam.

**Figure 3:**
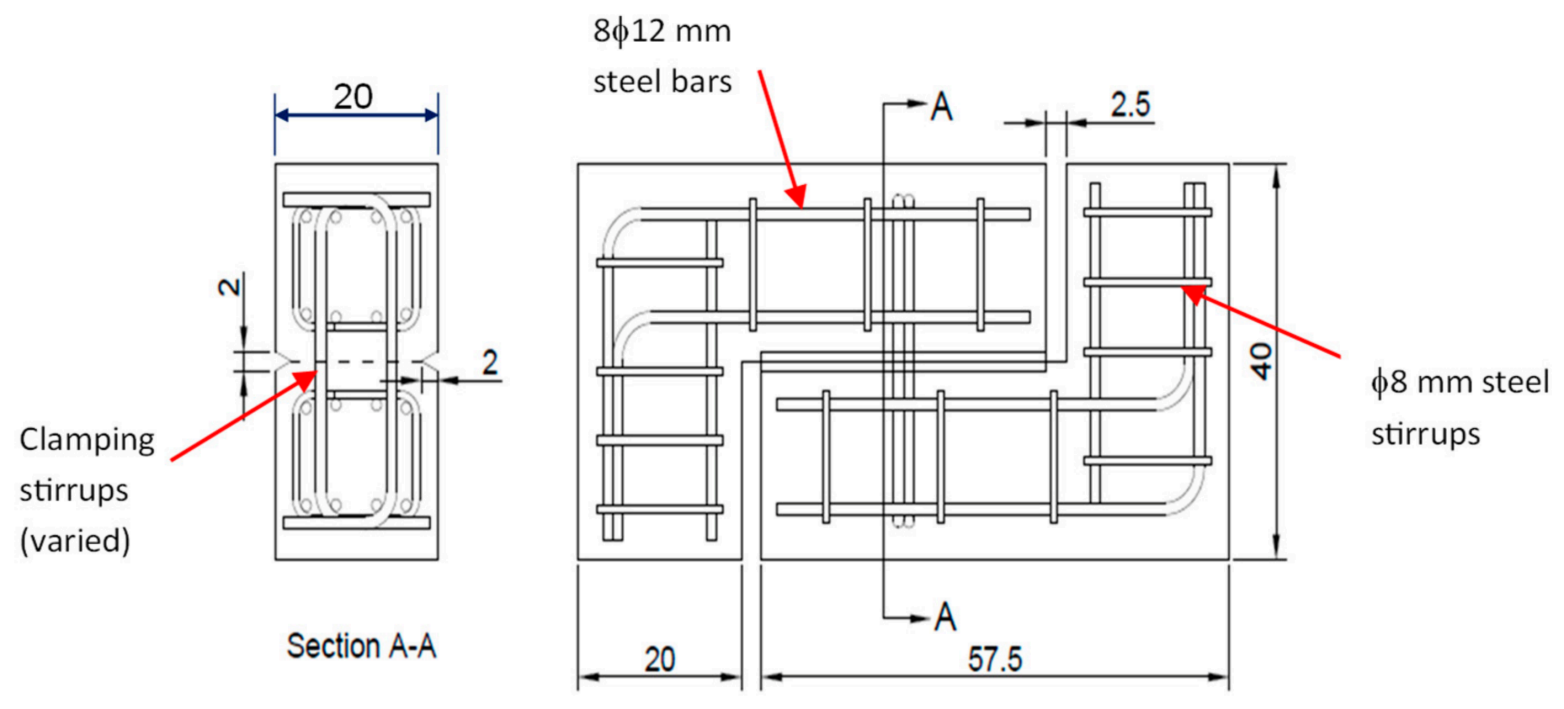
Dimensions and reinforcement details of push-off specimens (dimensions in cm).

**Figure 4:**
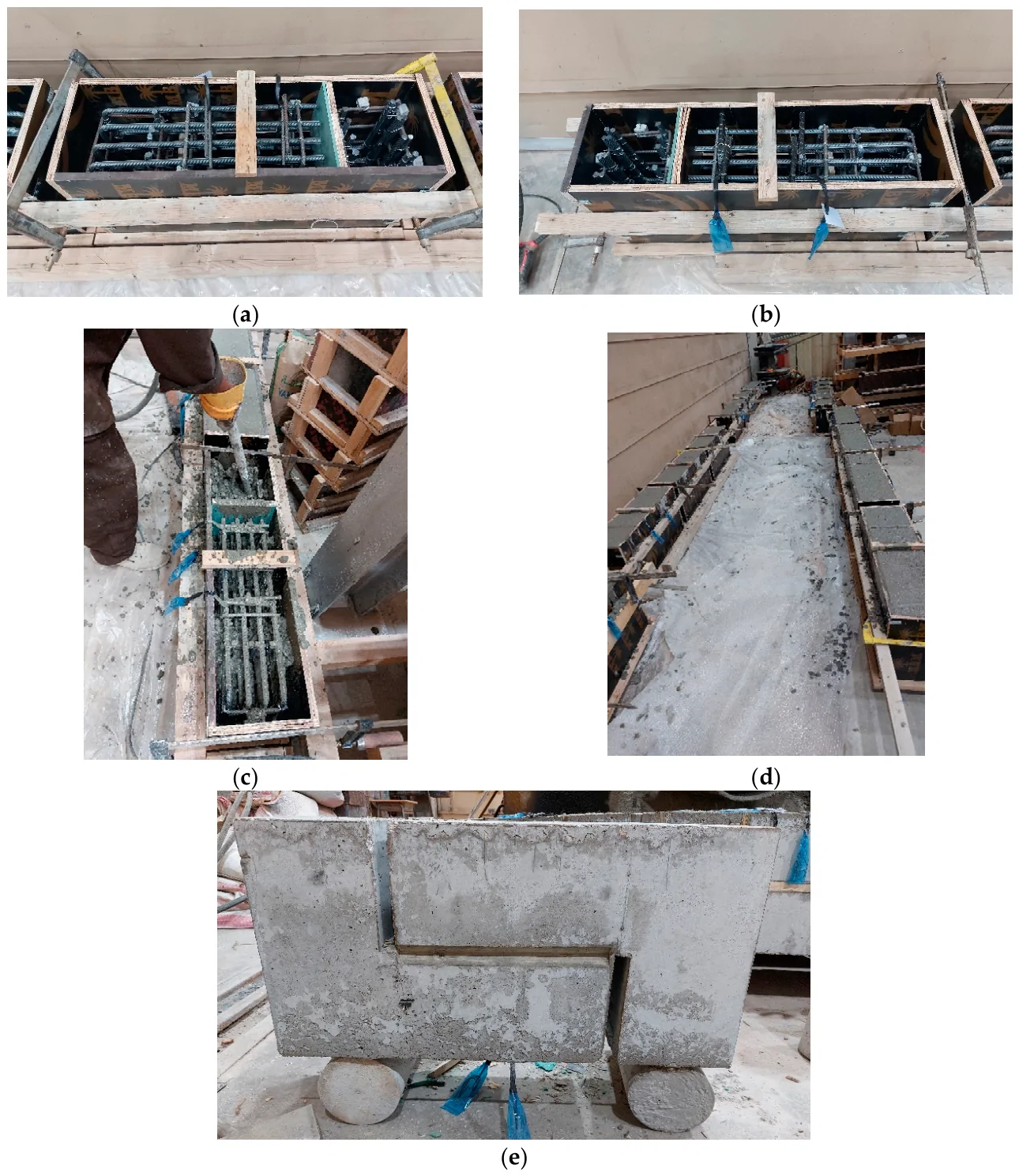
Stages of specimen construction: (**a**) Specimen reinforced with steel stirrups; (**b**) Specimen reinforced with GFRP stirrups; (**c**) Concrete casting; (**d**) Specimens after concrete casting; (**e**) Completed specimen.

**Figure 5:**
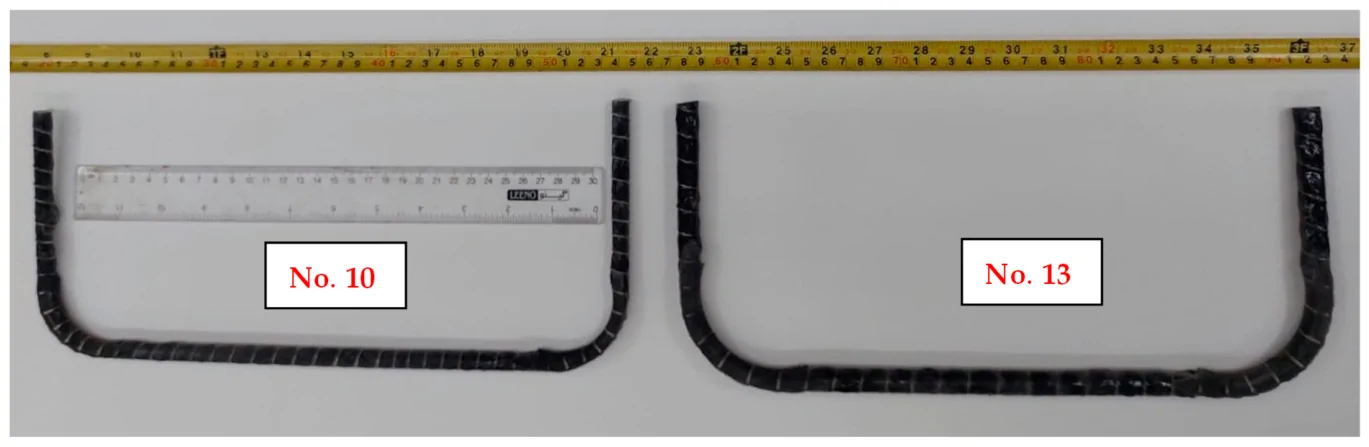
GFRP U-shaped stirrups.

**Figure 6:**
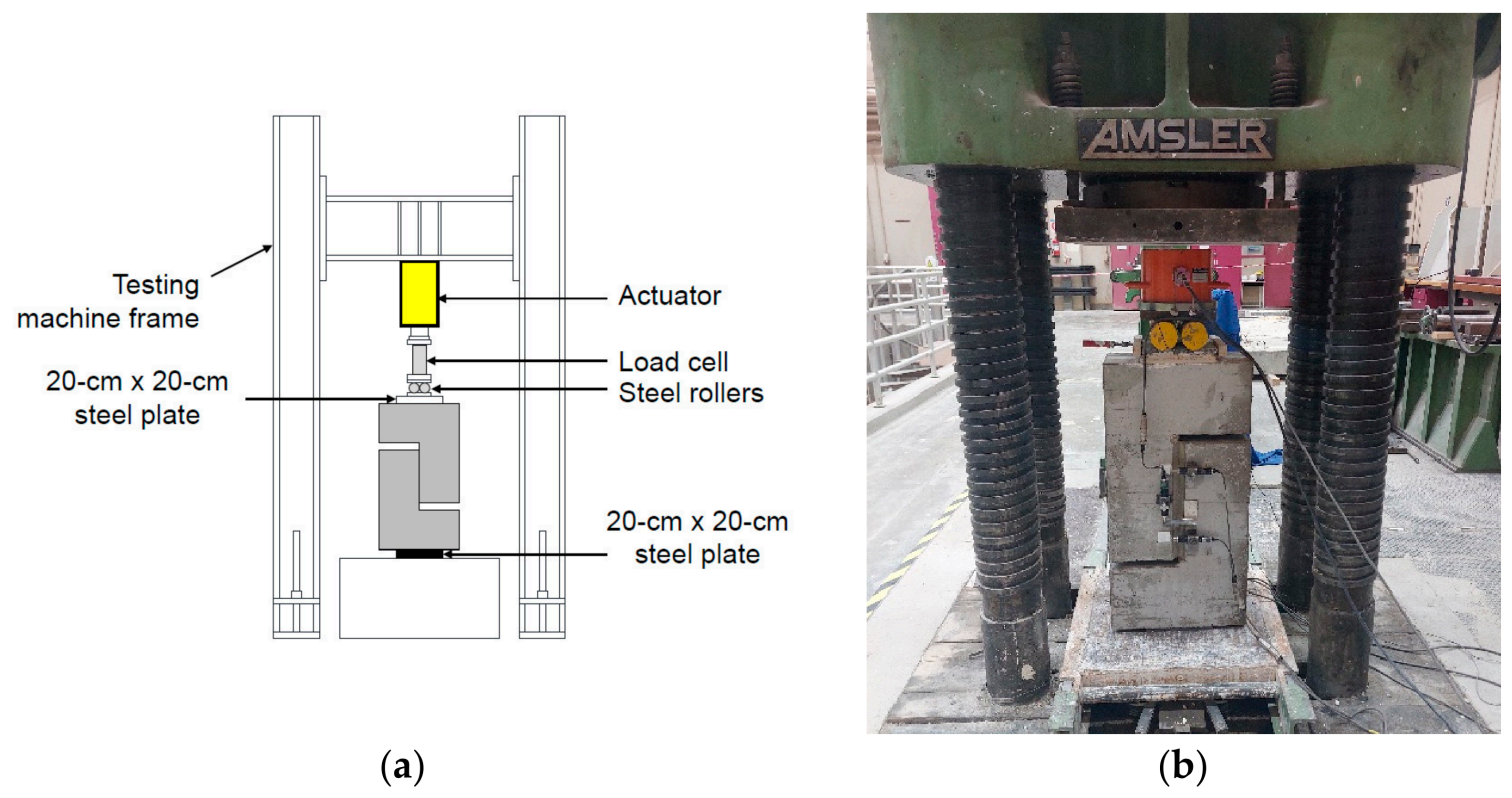
Details of push-off test setup: (**a**) Schematic representation; (**b**) Photographic view.

**Figure 7:**
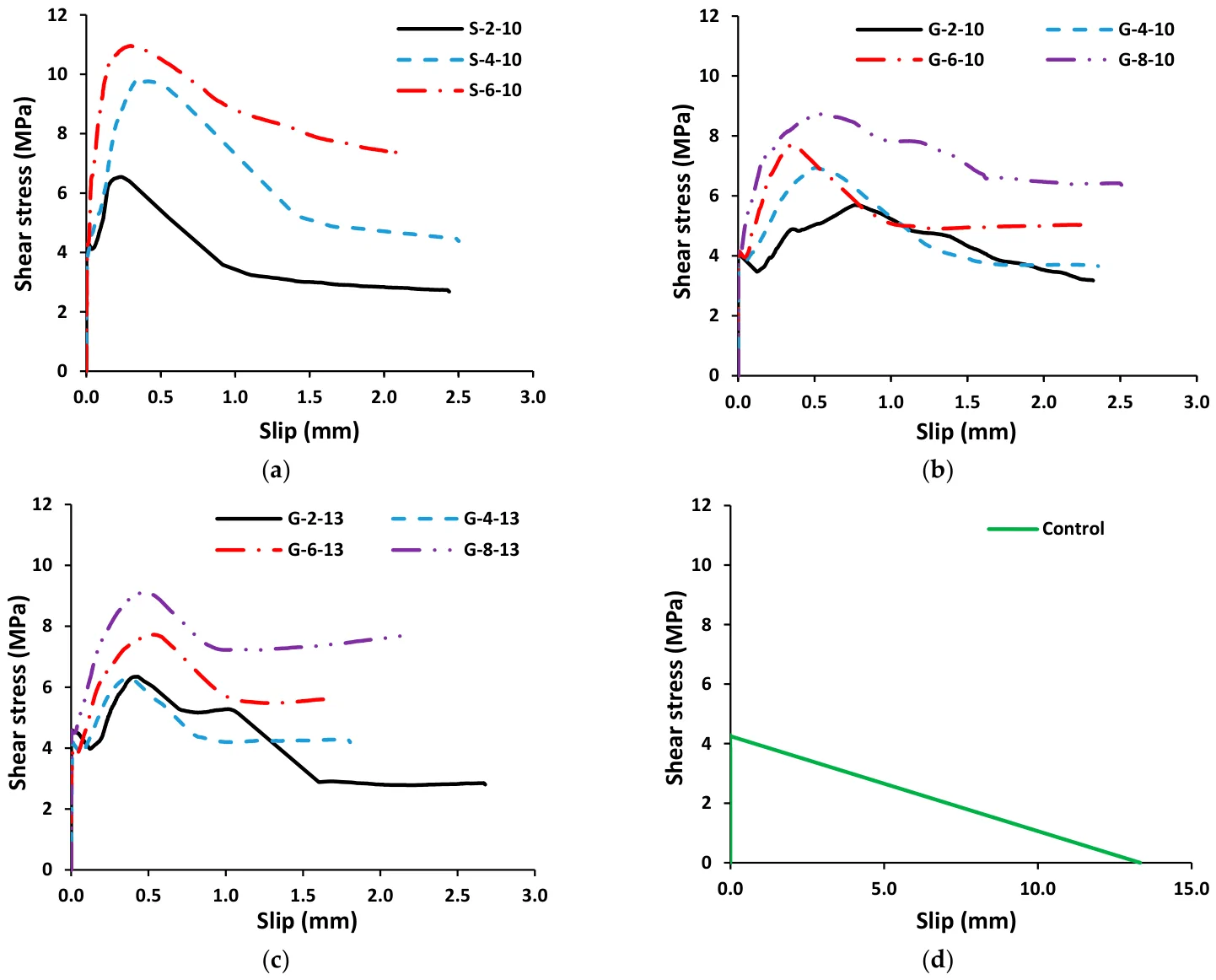
Shear stress-slip curves: (**a**) Specimens reinforced with steel stirrups; (**b**) Specimens reinforced with 10 mm–diameter GFRP stirrups; (**c**) Specimens reinforced with 13 mm–diameter GFRP stirrups; (**d**) Control specimen without clamping stirrups.

**Figure 8:**
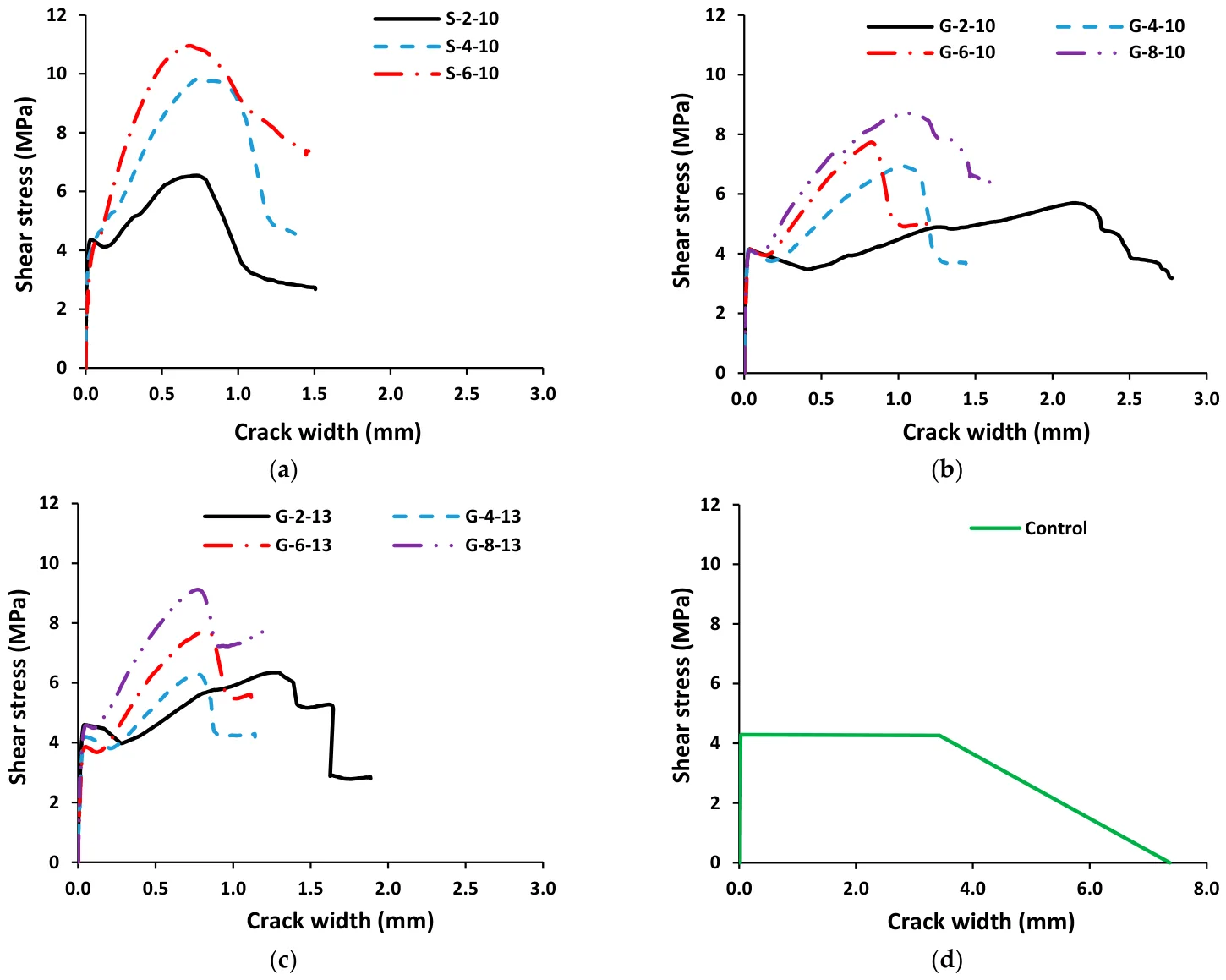
Shear stress-crack width curves: (**a**) Specimens reinforced with steel stirrups; (**b**) Specimens reinforced with 10 mm–diameter GFRP stirrups; (**c**) Specimens reinforced with 13 mm–diameter GFRP stirrups; (**d**) Control specimen without clamping stirrups.

**Figure 9:**
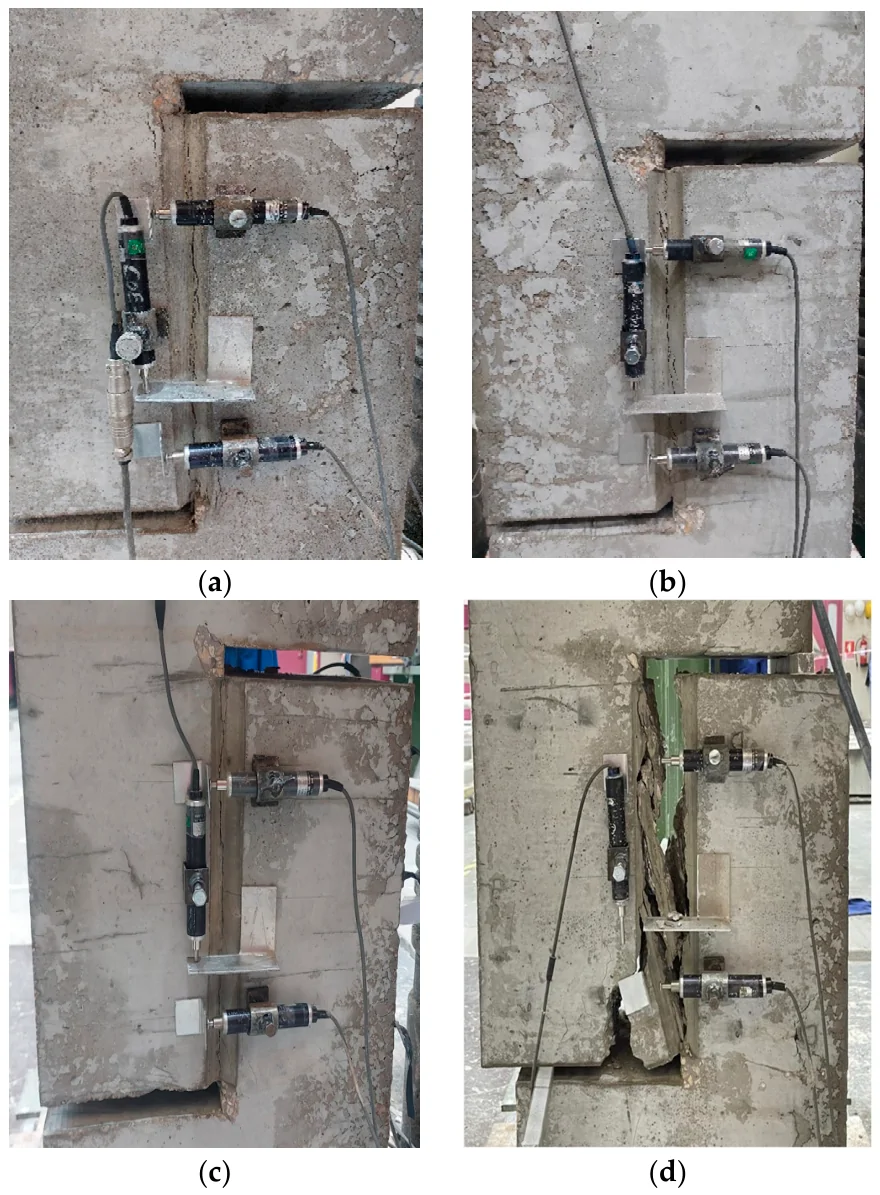
Typical mode of failure: (**a**) Specimen S–6–10; (**b**) Specimen G–6–10; (**c**) Specimen G–6–13; (**d**) Control specimen C.

**Figure 10:**
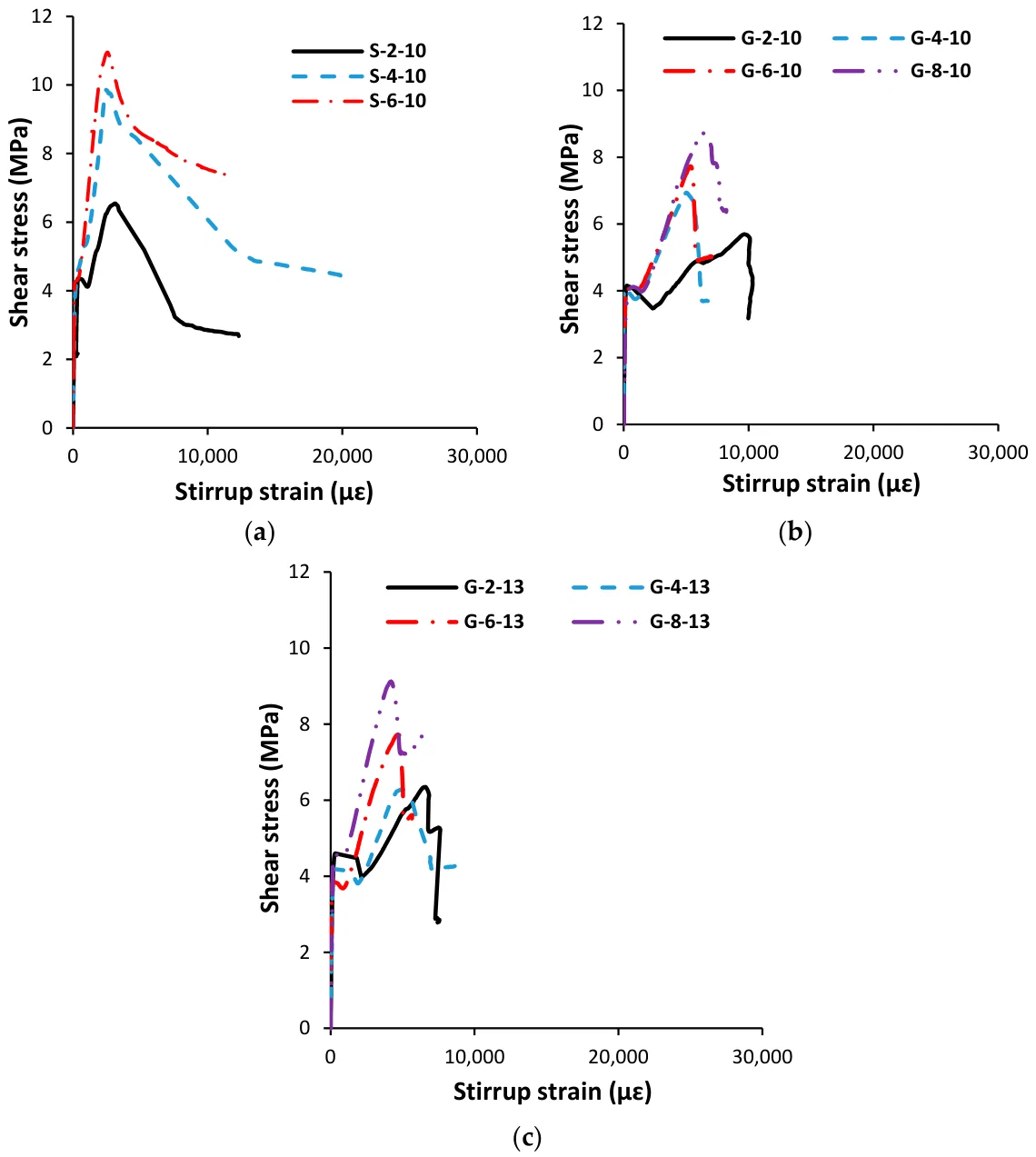
Shear stress-stirrup strain curves: (**a**) Specimens reinforced with steel stirrups; (**b**) Specimens reinforced with 10 mm–diameter GFRP stirrups; (**c**) Specimens reinforced with 13 mm–diameter GFRP stirrups.

**Figure 11:**
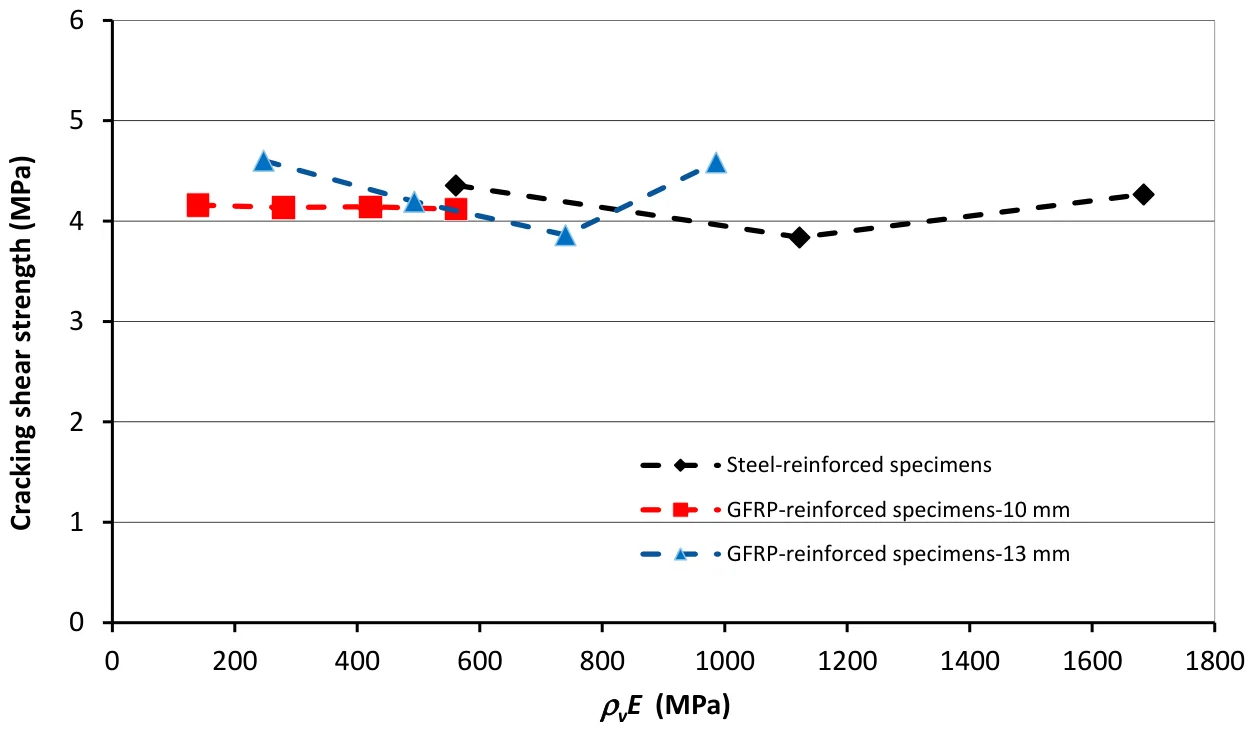
Variation in cracking shear strength with the axial stiffness of the clamping stirrups.

**Figure 12:**
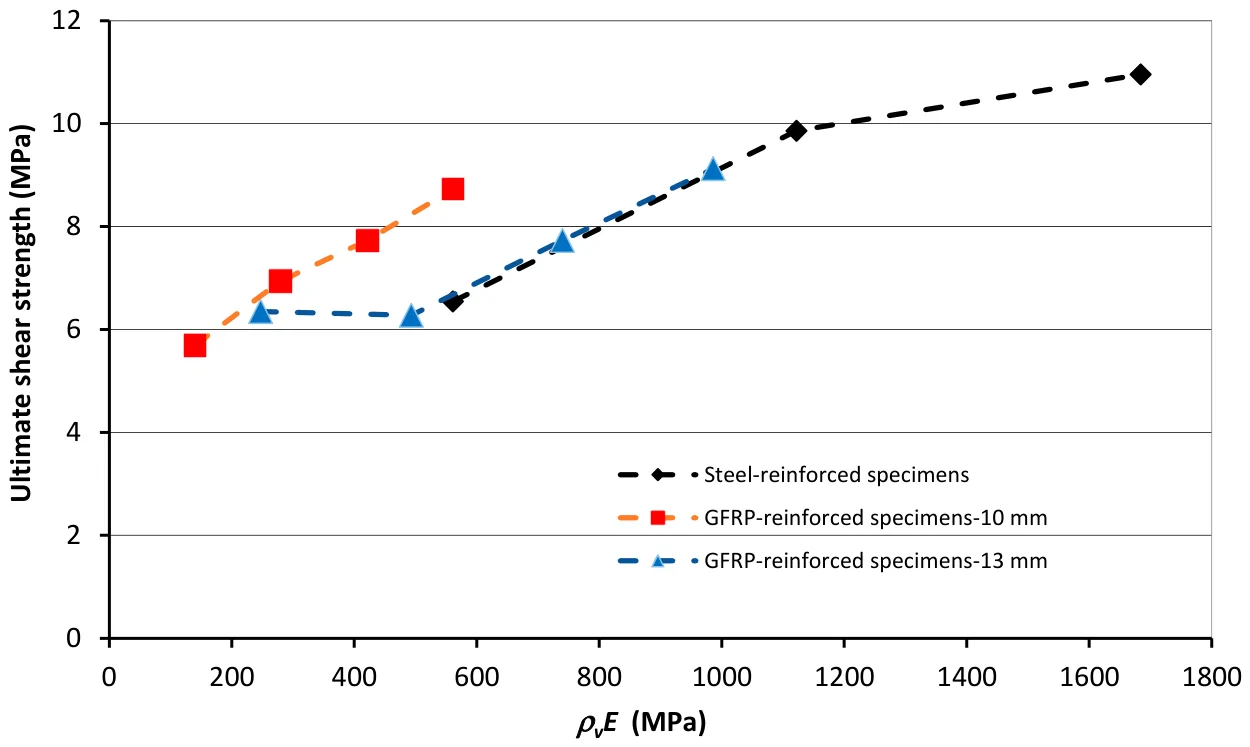
Variation in ultimate shear strength with the axial stiffness of the clamping stirrups.

**Figure 13:**
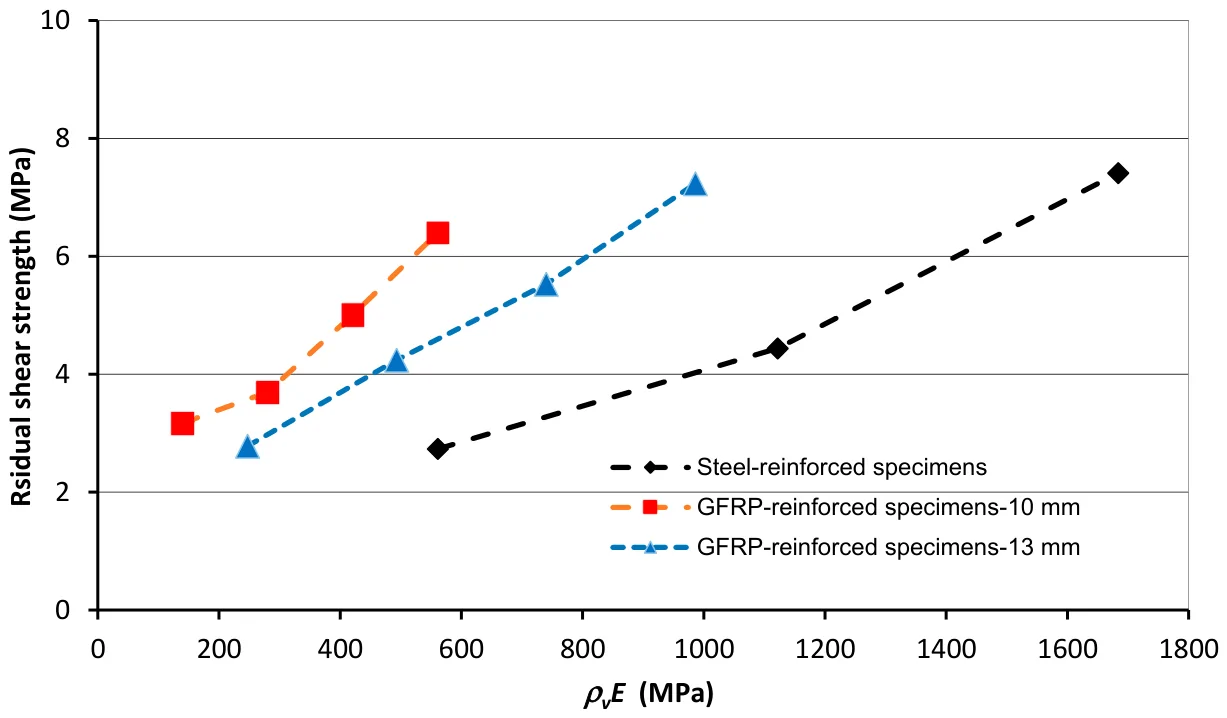
Variation in residual shear strength with the axial stiffness of the clamping stirrups.

**Figure 14:**
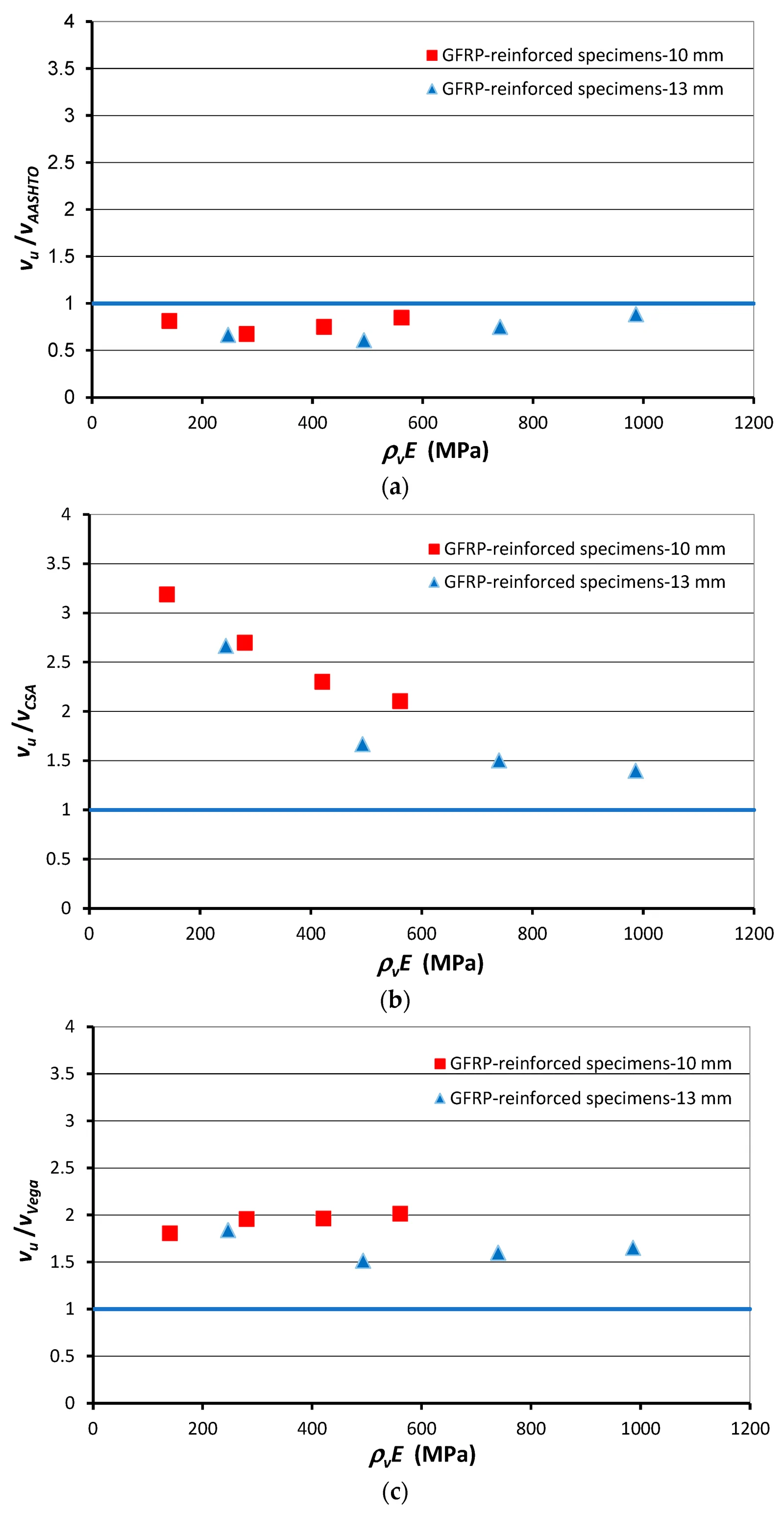
Comparison of experimental and predicted shear strength: (**a**) AASHTO predictions; (**b**) CSA S6 predictions; (**c**) Vega et al. predictions.

**Table 1:** Test matrix.

Specimen	Type of Clamping Stirrups	Diameter of Clamping Stirrups (mm)	Number of Stirrups Legs	Reinforcement Ratio, *ρ_v_* (%)	*ρ_v_E* (MPa)
C	N/A	N/A	N/A	N/A	N/A
S–2–10	Steel		2	0.28	561
S–4–10		10	4	0.56	1122
S–6–10			6	0.84	1684
G–2–10	GFRP		2	0.28	140
G–4–10		10	4	0.56	280
G–6–10			6	0.84	421
G–8–10			8	1.12	561
G–2–13	GFRP		2	0.47	247
G–4–13		13	4	0.95	493
G–6–13			6	1.4	740
G–8–13			8	1.9	986

**Table 2:** Reinforcement mechanical properties.

Reinforcement	Nominal Diameter (mm)	Bar Area (mm^2^)	Yield Strength (MPa)	Tensile Strength (MPa)	Modulus of Elasticity (GPa)
GFRP No. 10	9.5	71	–	1080	50
GFRP No. 13	12.7	129	–	1020	52
Steel 8	8	50	480	690	203
Steel 10	10	78.5	585	690	200
Steel 12	12	113	556	683	205

**Table 3:** Summary of experimental and calculated results.

Specimen	$f_{c}^{\prime }$ (MPa)	*v_cr_* (MPa)	*v_u_* (MPa)	*v_r_* (MPa)	$\frac{v_{r}}{v_{u}}$	Slip at *v_u_* (mm)	Crack Width at *v_u_* (mm)	Stirrup Strain at *v_u_* (με)
C	46.8	4.29	4.29	N/A	N/A	--	--	N/A
S–2–10		4.36	6.55	2.73	0.42	0.24	0.73	3250
S–4–10		3.84	9.86	4.44	0.45	0.35	0.74	2420
S–6–10		4.27	10.96	7.41	0.68	0.30	0.68	2660
G–2–10		4.16	5.69	3.17	0.56	0.77	2.15	9820
G–4–10		4.14	6.94	3.70	0.53	0.51	1.04	5036
G–6–10		4.14	7.73	5.01	0.65	0.37	0.83	5351
G–8–10		4.12	8.73	6.4	0.73	0.55	1.06	6210
G–2–13		4.60	6.35	2.78	0.44	0.43	1.29	6522
G–4–13		4.19	6.28	4.24	0.68	0.39	0.78	5066
G–6–13		3.86	7.73	5.53	0.72	0.52	0.82	4702
G–8–13		4.59	9.13	7.23	0.79	0.49	0.78	4120

**Table 4:** Comparison of experimental and predicted shear resistance of GFRP-reinforced specimens.

Specimen	*v_u_* (MPa)	Predicted Interface Shear Resistance					
		AASHTO		CSA S6		Vega et al.	
		*v_AASHTO_* (MPa)	*v_u/_v_AASHTO_*	*v_CSA_* (MPa)	*v_u/_v_CSA_*	*v_Vega_* (MPa)	*v_u/_v_Vega_*
G–2–10	5.69	7.0	0.81	1.79	3.19	3.15	1.81
G–4–10	6.94	10.3	0.67	2.57	2.7	3.55	1.96
G–6–10	7.73	10.3	0.75	3.36	2.3	3.94	1.96
G–8–10	8.73	10.3	0.85	4.14	2.11	4.33	2.01
G–2–13	6.35	9.53	0.67	2.38	2.67	3.45	1.84
G–4–13	6.28	10.3	0.61	3.76	1.67	4.14	1.52
G–6–13	7.73	10.3	0.75	5.14	1.5	4.83	1.6
G–8–13	9.13	10.3	0.89	6.52	1.4	5.52	1.65
Average			0.75		2.19		1.79
SD			0.1		0.64		0.19
CoV %			12.8		29.2		10.4

## Data Availability

Data from this study can be made available upon request.
